# Construction of a novel prognostic signature based on the composition of tumor-infiltrating immune cells in clear cell renal cell carcinoma

**DOI:** 10.3389/fgene.2022.1024096

**Published:** 2022-10-13

**Authors:** Weiwei Yu, Jiahui Lu, Cen Wu

**Affiliations:** ^1^ Department of Oncology, The Affiliated Wuxi People’s Hospital of Nanjing Medical University, Wuxi, Jiangsu, China; ^2^ Department of General Surgery, Rudong People’s Hospital, Nantong, Jiangsu, China

**Keywords:** TIICs, ccRCC, ImmuCellAI, prognosis, bioinformatics analysis

## Abstract

Emerging evidence has uncovered that tumor-infiltrating immune cells (TIICs) play significant roles in regulating the tumorigenesis and progression of clear cell renal cell carcinoma (ccRCC). However, the exact composition of TIICs and their prognostic values in ccRCC have not been well defined. A total of 534 ccRCC samples with survival information and TIIC data from The Cancer Genome Atlas (TCGA) dataset were included in our research. The ImmuCellAI tool was employed to estimate the abundance of 24 TIICs and further survival analysis explored the prognostic values of TIICs in ccRCC. In addition, the expression levels of immunosuppressive molecules (PDL1, PD1, LAG3, and CTLA4) in the high- and low-risk groups were explored. Various subtypes of TIICs had distinct infiltrating features and most TIICs exhibited dysregulated abundance between normal and tumor tissues. Moreover, specific kinds of TIICs had encouraging prognostic values in ccRCC. Further analysis constructed a 4-TIICs signature to evaluate the prognosis of ccRCC patients. Cox regression analyses confirmed the independent prognostic role of the signature in ccRCC. Moreover, immunosuppressive molecules, including PD1, LAG3, and CTLA4, were significantly upregulated in the high-risk group and predicted poor prognosis. However, PDL1 was not changed between high- and low-risk groups and could not predict poor prognosis. To sum up, our research explored the landscape of TIICs in ccRCC and established a novel 4-TIIC prognostic signature, which could effectively predict the prognosis for patients with ccRCC. Based on this signature, we also concluded that PDL1 may not predict prognosis in ccRCC.

## Introduction

Renal cell carcinoma (RCC) is the most widespread malignancy in the urinary system in adults, which takes up more than 90% of adult renal tumors. According to the tumor statistical data announced by the American Cancer Society (ACS), there will be 79,000 new cases of kidney and renal pelvis tumors, which cause about 13,920 cancer-related deaths in the United States (Siegel et al., 2022). Among all RCC subtypes, clear cell renal cell carcinoma (ccRCC) is the most common subtype, accounting for about 75%–85% of total RCC (Baldewijns et al., 2008). Usually, ccRCC is resistant to chemotherapy. Encouragingly, ccRCC has been confirmed to be sensitive to immunotherapies (Liu et al., 2022). With the recent breakthroughs in immunotherapy for solid tumors, several immune checkpoint inhibitors have been approved for treatment for ccRCC, which opens up new prospects for the treatment of ccRCC (Hammers, 2016). For example, nivolumab plus ipilimumab, pembrolizumab plus axitinib, avelumab plus axitinib, nivolumab plus cabozantinib, and atezolizumab plus bevacizumab received approval by the United States Food and Drug Administration (US-FDA) as first-line therapy for advanced ccRCC (Nocera et al., 2022).

For a long time, it has been considered that the response to immunotherapy is dependent on the abundance of tumor-infiltrating immune cells (TIICs) in the tumor microenvironment (TME) (Tahkola et al., 2018; Stenzel et al., 2020). With the significant advancement of high-throughput sequencing technologies and the development of computer-aided algorithms, it is possible to assess the abundance of TIICs based on transcriptome data. Several classic algorithms such as CIBERSORT (Newman et al., 2015), MCPcounter (Becht et al., 2016), TIMER (Li et al., 2017), xCell (Aran et al., 2017), and EPIC (Racle et al., 2017) have been widely applied to evaluate the abundance of TIICs (Liu et al., 2021; Li et al., 2022), estimate the status of tumors and even build immune-related diagnostic and prognostic signatures. In addition, Miao *et al.* constructed a highly accurate algorithm, which was named ImmuCellAI, which was used to estimate the tumor-infiltrating levels of TIICs (Miao et al., 2020), and expanding the scope of assessment of tumor-infiltrating levels of more T cell subsets. However, ImmuCellAI-dependent TIIC assessment has not been applied in ccRCC to describe the landscape of TIICs.

**FIGURE 1:**
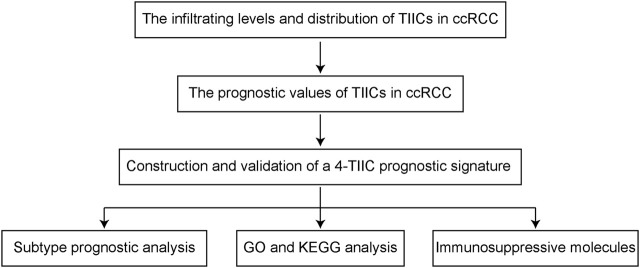
The flow chart of the current study.

In this research, based on the ImmuCellAI method and ccRCC transcriptome data from The Cancer Genome Atlas (TCGA) dataset, we performed a deep analysis of the effects of TIICs in ccRCC patients. As the result, we developed a novel prognostic signature based on four types of immune cells, including exhausted T cell (Tex), induced regulatory T cell (iTreg), T helper 17 cell (Th17), and central memory T cell (Tcm), which may exactly distinguish risk stratification in ccRCC patients. In addition, we also performed the subgroup analysis and enrichment analysis, and compared the expression of immunosuppressive molecules in various groups (Figure 1). To sum up, the current study established a novel 4-TIIC signature to predict prognosis and tumor immune microenvironment in ccRCC.

## Materials and methods

### Data acquisition

The survival data and RNA-seq data (IlluminaHiSeq) of ccRCC samples were obtained from the UCSC Xena website (https://xenabrowser.net/datapages/). TIIC data of ccRCC samples was downloaded from the ImmuCellAI website (http://bioinfo.life.hust.edu.cn/web/ImmuCellAI/) (Miao et al., 2020). Patients with missing or insufficient data were excluded from this research. Finally, 534 samples with completed survival and TIICs data were reserved for analysis.

### Least absolute shrinkage and selection operator cox analysis

In survival analysis, the overall survival (OS) event was set as the endpoint of observation. The 534 ccRCC samples were divided randomly into the training cohort (*n* = 267) and the testing cohort (*n* = 267). The training cohort was applied for prognostic signature construction, while the testing cohort and entire cohort were applied for validation of the established signature. To establish a TIIC-dependent prognostic signature, univariate Cox regression was firstly applied to screen the prognostic values of 24 TIIC abundance. The least absolute shrinkage and selection operator (LASSO) Cox regression model with ten-fold cross-validation was performed using R package “glmnet” and then applied for the further selection of prognostic TIICs and building prognostic signature. The risk score equaled the infiltrating abundance of TIICs multiplied by corresponding regression coefficients. Kaplan-Meier curves and log-rank test were applied to assess the difference in OS by setting the median value of the risk score as the cut-off value. Univariate and multivariable Cox analyses were performed to explore the independent prognostic value of the risk score in combination with clinic-pathological features.

### Identification of differentially expressed genes

R language was applied to screen differentially expressed genes (DEGs) between low-risk and high-risk samples using the R package “limma.” The thresholds of extracting DEGs were as follows: |log2 [fold change (FC)]| > 1, and *p* < 0.01. Volcano plots were drawn using the SangerBox tool (Shen et al., 2022). Next, Database for Annotation, Visualization and Integrated Discovery (DAVID, https://david.ncifcrf.gov/) was employed to perform Gene Ontology (GO) and Kyoto Encyclopedia of Genes and Genomes (KEGG) analyses of DEGs. The human genome (*Homo sapiens*) was selected as the background variable. The top five terms or terms with *p* value <0.05 were exhibited as results.

### Collection of clear cell renal cell carcinoma specimens

The tumor tissue microarray of ccRCC (TMA, HKidE180Su02) was purchased from Outdo BioTech (Shanghai, China). The HKidE180Su02 cohort contained 150 ccRCC and 30 para-tumor samples. Detailed clinic-pathological information on the TMA and follow-up data were provided by Outdo BioTech, which was exhibited in Supplementary Table S1. Ethical approval was granted by the Clinical Research Ethics Committee in Outdo Biotech (Shanghai, China).

### Immunohistochemistry staining and semi-quantitative assessment

Immunohistochemistry (IHC) staining was conducted on the HKidE180Su02 TMA according to the standardized procedures. The sections were then washed with xylene for three 5-min. The sections were rehydrated by successive washes in 100%, 90%, and 70% graded ethanol. Hydrogen peroxidase was used to block endogenous peroxidase activity for 20 min. The antigen retrieval solution is EDTA. The primary antibody used in our research was anti-PD-L1 (Ready-to-use, Cat. GT2280, GeneTech). Antibody staining was visualized with DAB and hematoxylin counterstain, and stained TMA was captured using Aperio Digital Pathology Slide Scanners. The stained TMA was independently evaluated by two pathologists. Expression levels of PD-L1 in tumor cells were semi-quantitatively assessed by estimating the immunoreactivity score (IRS) (Mei et al., 2020; Mei et al., 2021). Briefly, the percentage of positively stained cells was scored as 0–4: 0 (<5%), 1 (6%–25%), 2 (26%–50%), 3 (51%–75%) and 4 (>75%). The staining intensity was scored as 0–3: 0 (negative), 1 (weak), 2 (moderate), and 3 (strong). The IRS equals the percentages of positive cells multiplied by staining intensity.

### Statistical analysis

R 4.0.2 and GraphPad Prism 8.0 were applied as the main tools for the statistical treatment and figure display. The difference between the two groups was mostly detected by Student’s t-test. Correlation analysis was conducted by Pearson’s test. The LASSO Cox regression model was applied to further screen the prognostic TIICs using R package “glmnet.” Kaplan-Meier survival plots were built to compare the difference in OS using the log-rank test. Univariate and multivariate Cox regression models were used to calculate the hazard ratio (HR) of the risk score to clinic-pathological features for OS. For all analyses, *p* value <0.05 was deemed to be statistically significant and labeled with *p < 0.05, **p < 0.01, and ***p < 0.001.

## Results

### The infiltrating levels and distribution of tumor-infiltrating immune cells in clear cell renal cell carcinoma

**FIGURE 2:**
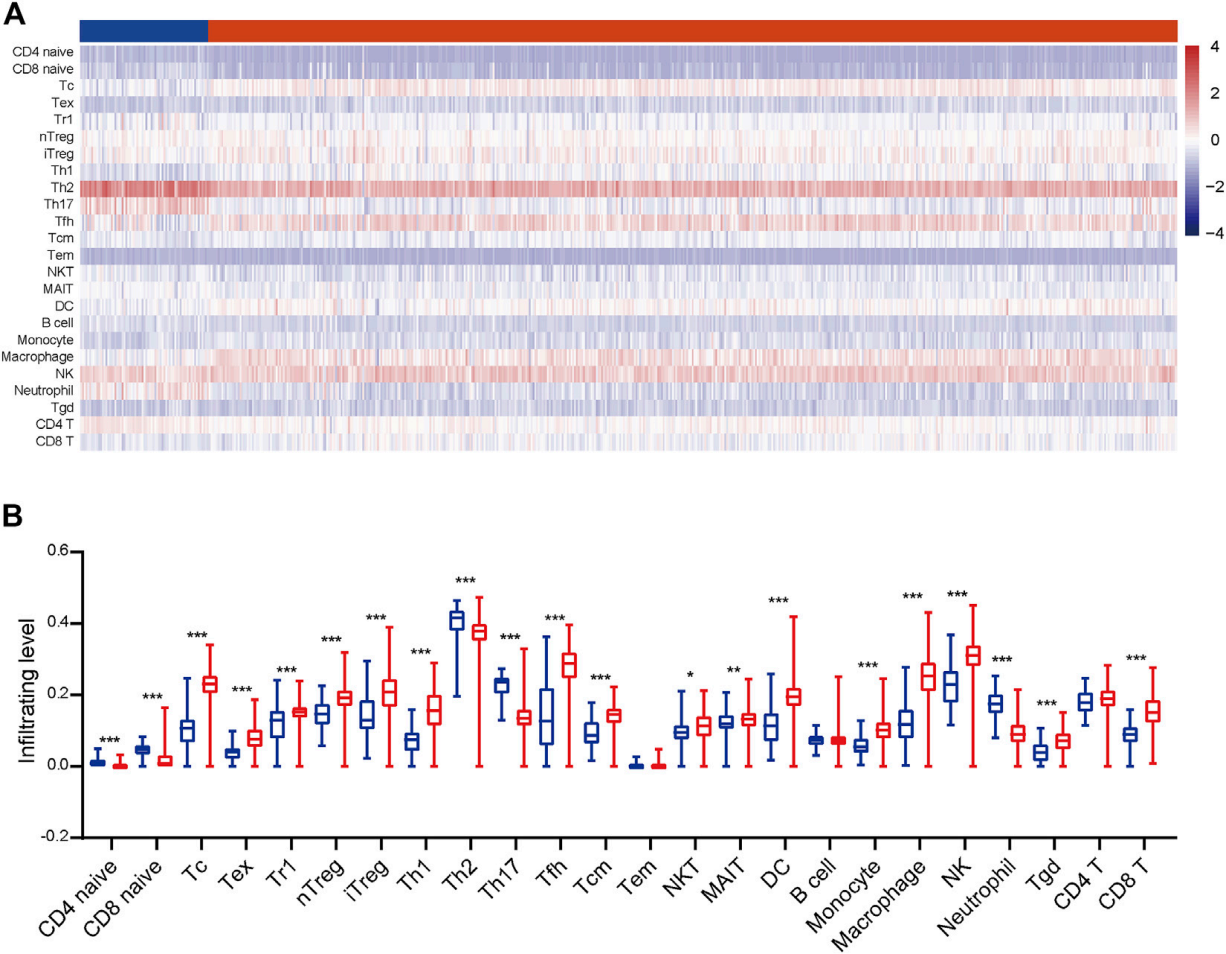
The distribution of TIICs in ccRCC. **(A)** The heatmap of the 24 TIICs abundance based on TCGA data. Red represents high abundance, and blue represents low abundance. **(B)** The infiltrating abundance of 24 TIICs in normal and tumor tissues. Significance was calculated with Student’s t-test. *p < 0.05, **p < 0.01, ***p < 0.001.

**FIGURE 3:**
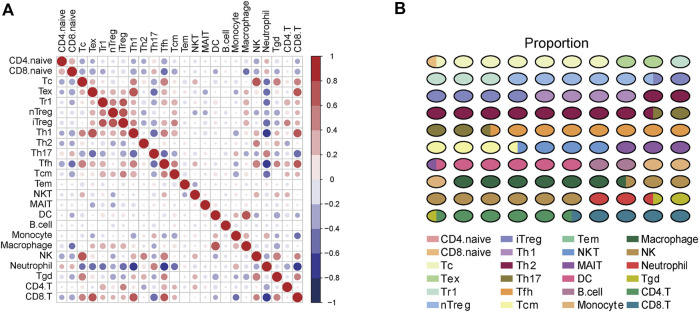
The correlation and proportion of TIICs in ccRCC. **(A)** Correlation matrix of 24 TIICs in ccRCC samples. Red represents positive correlation, and blue represents negative correlation. Significance was calculated with Pearson correlation analysis. **(B)** The proportion of 24 TIICs in ccRCC.

To gain a comprehensive insight into TIICs in ccRCC, the ImmuCellAI was used to assess TIICs levels in ccRCC samples from the TCGA dataset. A heatmap was plotted to exhibit 24 immune cells abundance in normal and tumor samples (Figure 2A). We further compared the level of each TIIC in ccRCC tissues. As the results exhibited, most TIICs were significantly dysregulated in ccRCC tissues. Cytotoxic T cell (Tc), Tex, type 1 regulatory T cell (Tr1), etc. were significantly enriched, while CD4^+^ naive cell, CD8^+^ naive cell, Th2, etc. were notably decreased in ccRCC tissues compared with the normal tissue (Figure 2B). In addition, the correlation between TIICs in ccRCCs was evaluated to understand the potential relationships among different immune cell types. It was uncovered that the fractions of several types of immune cells had a correlation with each other in TCGA (Figure 3A). Subsequently the proportion of various TIICs in ccRCC samples. The results exhibited that CD4^+^ naive cell had the lowest abundance level, while Th2 had the highest abundance level in ccRCC (Figure 3B). Taken together, given most kinds of TIICs being dysregulated in ccRCC, we speculated that infiltrating TIICs might play significant roles in mediating ccRCC progression.

### The prognostic values of tumor-infiltrating immune cells in clear cell renal cell carcinoma

**FIGURE 4:**
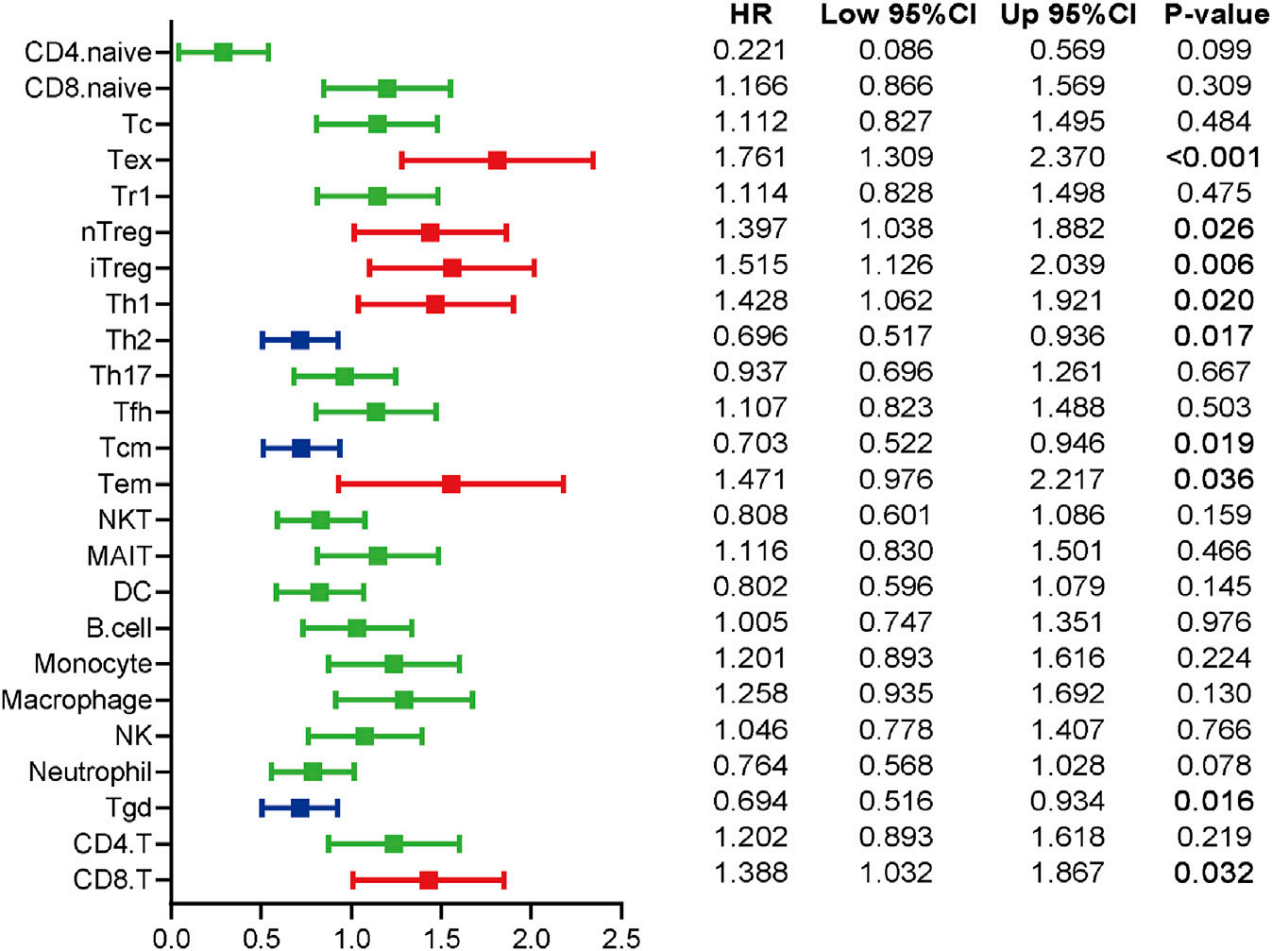
The prognostic values of TIICs in ccRCC patients. Overview of Kaplan-Meier analysis for the prognostic values of 24 TIICs in ccRCC. Red represents risky factor, and blue represents protective factor. Significance was calculated with log-rank analysis.

The patients were divided into two groups at the cut-off value of the median infiltrating levels to assess the prognostic values of TIICs in ccRCC. The results exhibited that several kinds of TIICs had notableprognostic values in ccRCC patients (Figure 4). Patients with higher levels of Tex, natural regulatory T cell (nTreg), Th1, effector memory T cell (Tem), and CD8^+^ T cell had notably worse OS, while high infiltrating levels of Th2, Tcm, and gamma delta T cell (Tgd) predicted better prognosis in ccRCC patients (Figure 4). Overall, these findings revealed that several TIICs have specific prognostic values, which could be used as prognostic indicators in ccRCC.

### Construction and validation of a 4-tumor-infiltrating immune cells prognostic signature

In view of the notable prognostic values of TIICs, we tried to establish a TIIC-associated prognostic signature. Firstly, we conducted univariate Cox regression to initially screen TIICs with significant effects on the prognosis of ccRCC in the training cohort and thus five types of TIICs were extracted (Supplementary Table S2). Secondly, LASSO Cox analysis was performed to further detect the effective TIICs and construct prognostic signature (Supplementary Figures S1A,B). Six TIICs were identified and then used to construct a prognostic signature. We successfully constructed a 4-TIIC signature (2.307*Tex+1.586*iTreg-0.556*Th17-3.679*Tcm) to evaluate the prognosis of ccRCC patients based on the tumor-infiltrating levels of these four TIICs and their regression coefficients (Supplementary Figure S1C).

**FIGURE 5:**
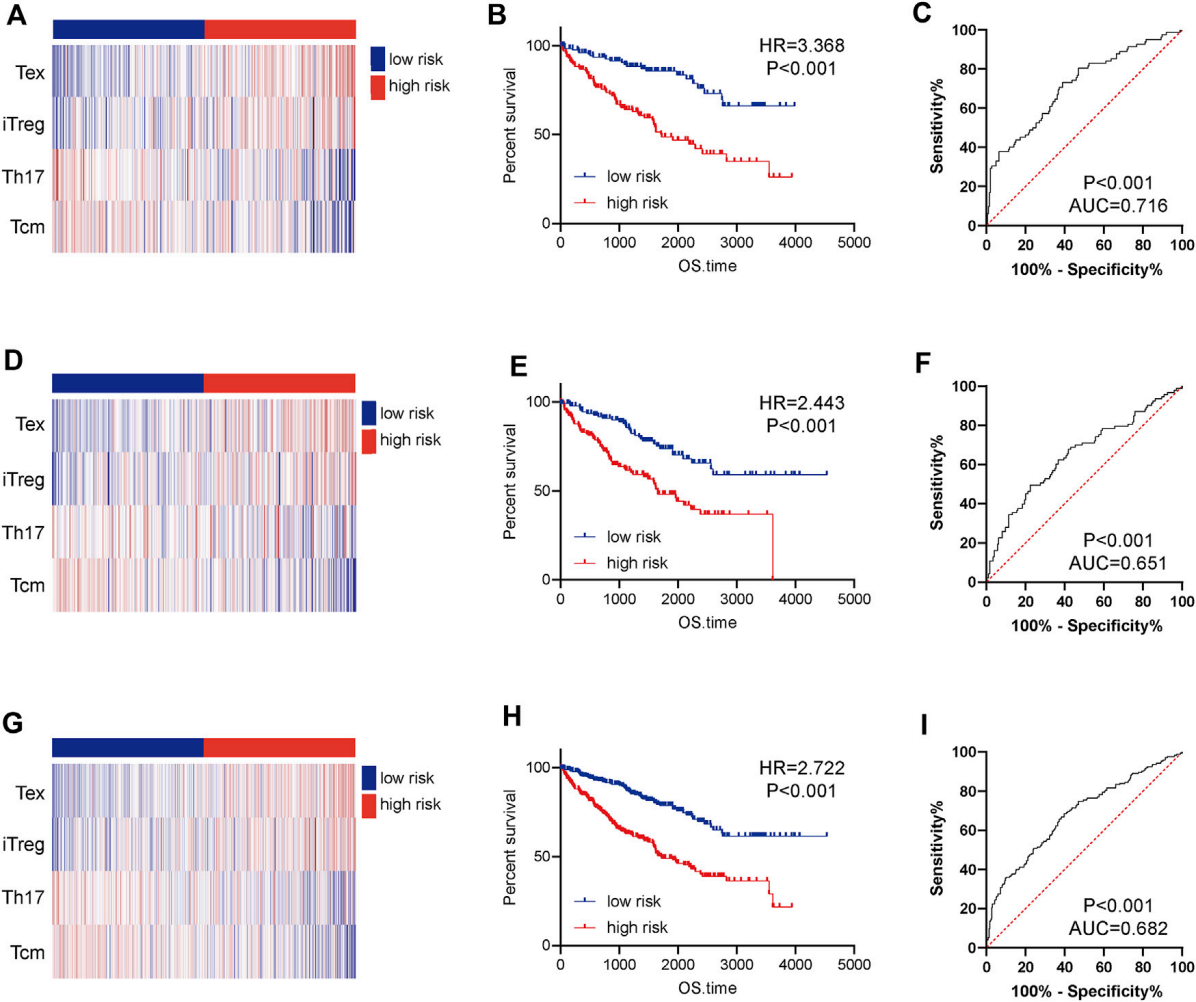
Construction and validation of a 4-TIIC prognostic signature for ccRCC. The heatmap showed the infiltrating abundance of four TIICs in the low and high-risk groups in **(A)** the training cohort, **(D)** the testing cohort, and **(G)** the entire cohort. Patients in the high-risk group exhibited worse prognosis than those in the low-risk group in **(B)** the training cohort, **(E)** the testing cohort, and **(H)** the entire cohort. Significance was calculated with log-rank analysis. ROC analysis of the 4-TIIC signature in **(C)** the training cohort, **(F)** the testing cohort, and **(I)** the entire cohort.

Based on the median value of the risk score, the patients in the training cohort were assigned into low- and high-risk groups. The distributions of four TIICs in the training cohort were exhibited in Figure 5A. Kaplan-Meier analysis showed that patients with high-risk showed notably worse OS compared with those with low risk (Figure 5B). In addition, the AUC in the training cohort was 0.716, showing a highly exact identification capability (Figure 5C). To test the prognostic value of the immune signature, the testing cohort and the entire cohort were employed. The distributions of four TIICs in the testing and entire cohorts were shown in Figures 5D,G. Similar to the training cohort, the prognosis of high-risk patients was notably worse than those of the low-risk patients in both testing and entire cohorts (Figures 5E,H). ROC analysis confirmed the satisfactory prognostic accuracy of the 4-TIIC signature in testing and entire cohorts as well (Figures 5F,I). Moreover, univariate and multivariate Cox analysis confirmed that this prognostic signature could independently predict the prognosis for ccRCC patients (Table 1). Totally, the novel signature could effectively predict the prognosis for ccRCC patients.

**TABLE 1:** Univariate and multivariate analysis of survival factors in patients with ccRCC.

Characteristics	Univariate analysis			Multivariate analysis		
	HR	95% CI	*p*-value	HR	95% CI	*p*-value
Gender	0.944	0.694–1.284	0.714			
Age	1.029	1.016–1.042	**<0.001**	1.027	1.013–1.041	**<0.001**
Grade	2.286	1.868–2.798	**<0.001**	1.292	1.021–1.635	**0.033**
Clinical stage	1.886	1.655–2.150	**<0.001**	1.623	1.394–1.889	**<0.001**
Risk-score	59.527	23.812–148.813	**<0.001**	10.725	3.971–28.967	**<0.001**

### Subtype prognostic analysis of the 4-tumor-infiltrating immune cells signature in clear cell renal cell carcinoma

**FIGURE 6:**
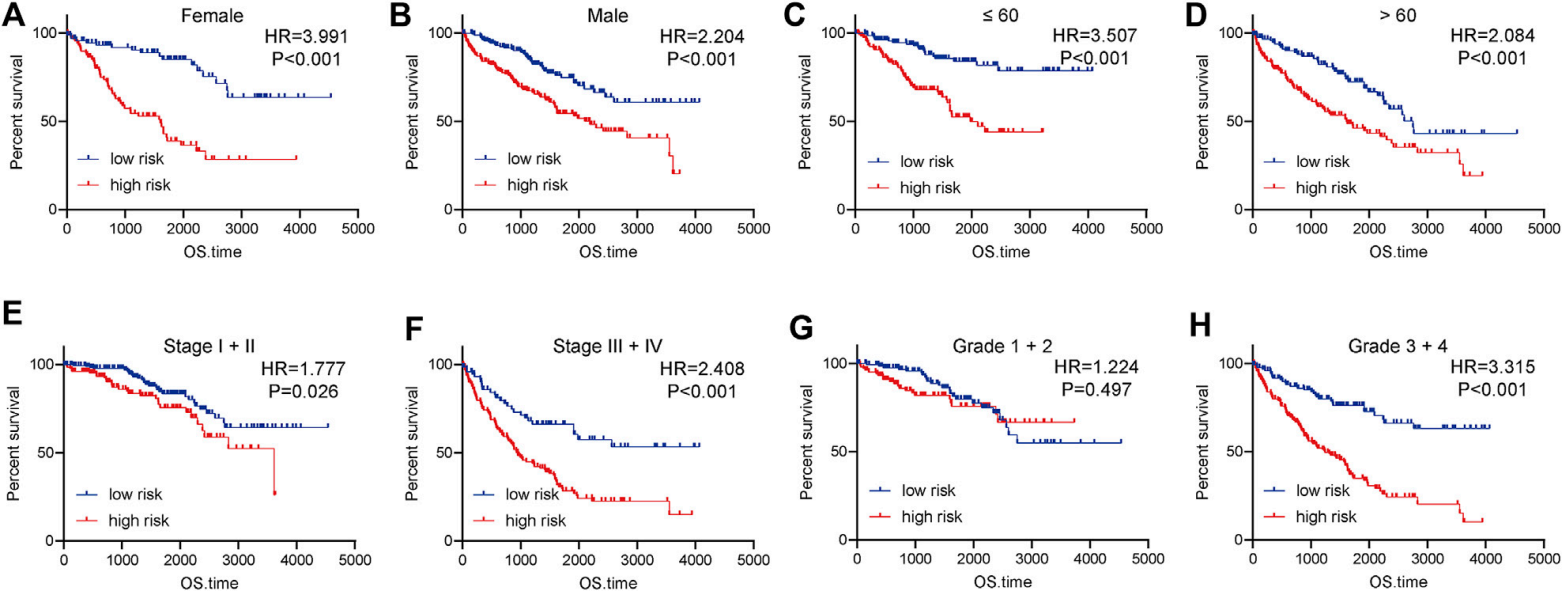
Subgroup analysis of the prognostic value of the risk score in ccRCC. **(A,B)** The prognostic value of the risk score in female and male patients. **(C,D)** The prognostic value of the risk score in patients with different ages. **(E,F)** The prognostic value of the risk score in patients at different clinical stages. **(G,H)** The prognostic value of the risk score in patients with various differentiated grades. Significance was calculated with log-rank analysis.

We next evaluate the prognostic value of the 4-TIIC signature in ccRCC patients with different clinic-pathological features in the entire cohort. The risk score could confirm satisfactory prognostic discrimination in patients with different genders and ages (Figures 6A–D). In terms of the clinical stage, the risk score was lowly correlated with OS in stage I and II patients, whereas in stage III and IV patients, the OS of patients with a higher risk score was obviously worse than that of the low expression group (Figures 6E,F). Furthermore, subgroup analysis by differentiation grade exhibited that the risk score did not have an association with OS in grade I and grade II patients, but in the patients with worse tumor differentiation, the OS of grade III and IV patients with high-risk score was notably worse than that with low-risk score (Figures 6G,H). Interestingly, subgroup analysis revealed that the prognostic value of 4-TIIC signature was more notable in patients with higher TNM stages and poorer differentiated grades.

### Expression levels of immunosuppressive molecules in various groups

To further understand the progression of ccRCC in term of the 4-TIIC signature, we exacted DEGs between two groups. A total of 194 genes were upregulated in the high-risk groups and 37 genes were upregulated in the low-risk group. We next extracted DEGs for enrichment analysis. The results showed that genes upregulated in the high-risk groups were associated with immune-related functions, and genes upregulated in the low-risk groups were relatively scattered (Table 2).

**TABLE 2:** GO and KEGG analysis of DEGs between low- and high-risk groups.

Category	Term	Count	*p* value
**Upregulated genes in high-risk group**			
BP	acute-phase response	11	<0.001
BP	positive regulation of peptide hormone secretion	4	<0.001
BP	protein polymerization	4	<0.001
BP	innate immune response	15	<0.001
BP	fibrinolysis	4	<0.001
CC	extracellular region	42	<0.001
CC	blood microparticle	9	<0.001
CC	plasma membrane	61	<0.001
CC	high-density lipoprotein particle	5	<0.001
CC	extracellular space	30	<0.001
MF	receptor binding	11	0.001
MF	serine-type endopeptidase inhibitor activity	6	0.001
MF	structural molecule activity	7	0.004
MF	cell adhesion molecule binding	4	0.018
MF	protein binding	112	0.024
KEGG	Cytokine-cytokine receptor interaction	10	0.001
KEGG	Complement and coagulation cascades	4	0.035
KEGG	Cell adhesion molecules	5	0.044
**Upregulated genes in low-risk group**			
BP	kidney development	4	0.001
BP	phospholipase C-activating G-protein coupled receptor signaling pathway	3	0.005
BP	signal transduction	7	0.014
BP	response to bacterium	3	0.016
BP	intracellular steroid hormone receptor signaling pathway	2	0.018
CC	plasma membrane	17	0.002
CC	integral component of plasma membrane	9	0.002
CC	extracellular exosome	10	0.006
CC	apical plasma membrane	4	0.020
CC	synapse	4	0.040
MF	peptide binding	3	0.005
MF	monocarboxylic acid transmembrane transporter activity	2	0.027
MF	steroid binding	2	0.047
MF	zinc ion binding	5	0.048
KEGG	Neuroactive ligand-receptor interaction	5	0.006
KEGG	Regulation of lipolysis in adipocytes	3	0.006
KEGG	PPAR signaling pathway	3	0.010
KEGG	Calcium signaling pathway	4	0.013
KEGG	Vascular smooth muscle contraction	3	0.031

**FIGURE 7:**
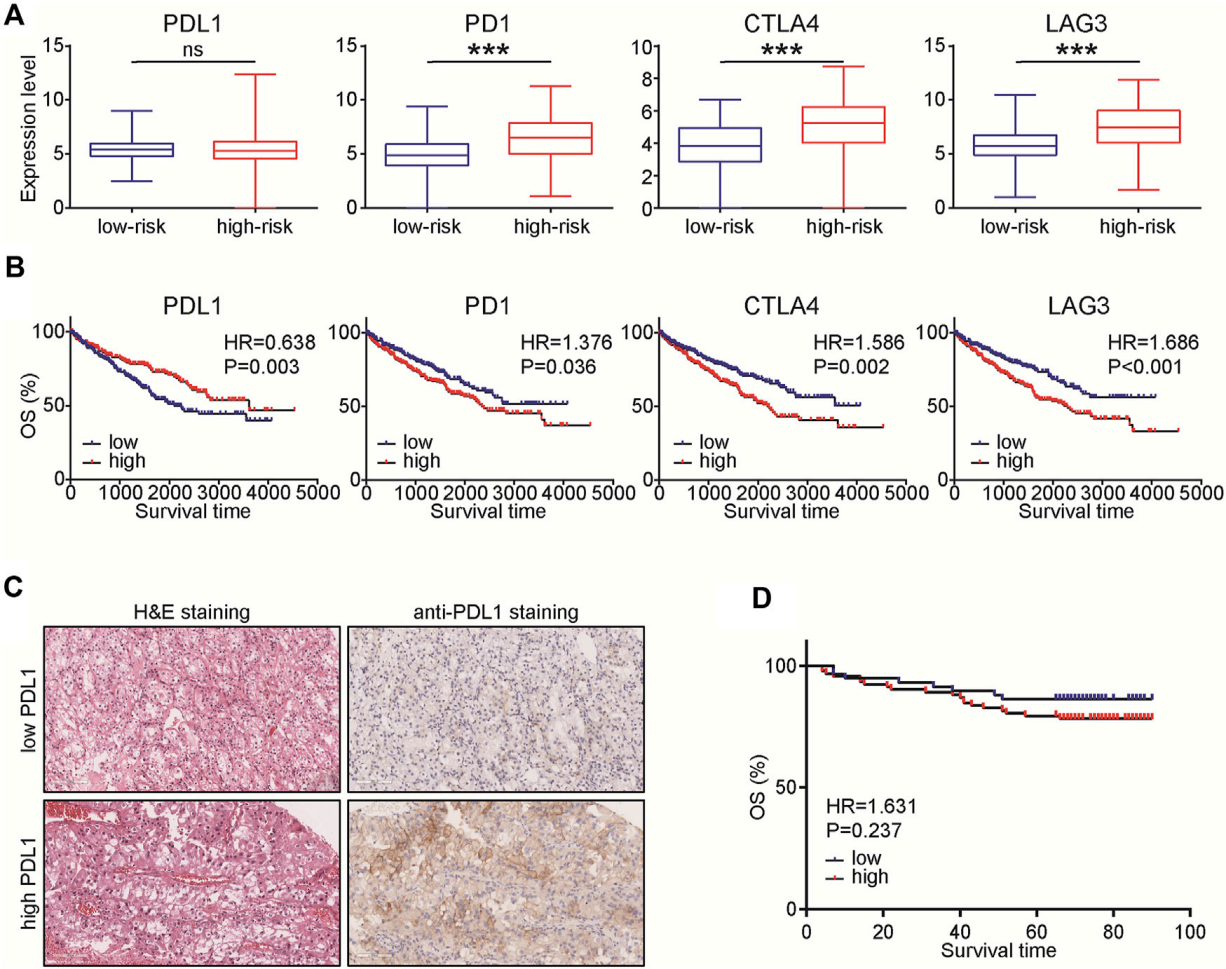
Expression levels of immunosuppressive molecules in various groups. **(A)** Expression levels of PDL1, PD1, CTLA4, and LAG3 in ccRCC with low- and high-risk. Significance was calculated with Student’s t-test. ***p < 0.001. **(B)** Prognostic values of PDL1, PD1, CTLA4, and LAG3 in ccRCC. Significance was calculated with log-rank analysis. **(C)** Representative images revealing PDL1 expression in ccRCC samples using anti-PDL1 staining. Magnification, × 200. **(D)** Prognostic values of PDL1 in 150 ccRCC patients. Significance was calculated with log-rank analysis.

Given the four types of immune cells in the prognostic signature, we speculated that ccRCC samples in the high-risk group may be in the immunosuppressive status. Thus, we compared the expression of several immunosuppressive immune checkpoints, including PDL1, PD1, LAG3, and CTLA4 in ccRCC samples in low- and high-risk groups. The results showed that PDL1 was not changed between high- and low-risk groups and could predict well prognosis, but PD1, LAG3, and CTLA4 were significantly upregulated in samples with high-risk (Figures 7A,B). Considering the unusual result, we further explored the prognostic value of PDL1 in ccRCC. A total of 150 ccRCC patients were involved and the result showed that PDL1 was not a prognostic factor in ccRCC patients (Figures 7C,D). Taken together, based on the 4-TIIC signature, we found that PDL1 might not be a prognostic biomarker in ccRCC.

## Discussion

A previous study presented by Chevrier et al. (2017) built an immune atlas of ccRCC and found 17 tumor-associated macrophage subtypes and 22 T cell subtypes. Besides, they suggested that immune cell compositions have obvious associations with progression-free survival (PFS) of ccRCC patients. Moreover, it was found in a study that ccRCC relapse after surgery has an association with decreased T cell infiltration level (Ghatalia et al., 2019). Authoritative research indicated a close correlation of immune cell infiltration with ccRCC progression and its value in prognosis and relapse prediction (Giraldo et al., 2017). In the current study, we systematically summarized the infiltrating levels and features of TIICs in ccRCC tissues by using the ImmuCellAI algorithm. Most dysregulated TIICs were found in ccRCC, which might play a significant role in ccRCC progression.

Growing numbers of research suggested that the abundance of TIICs in TME was associated with patient’s prognosis (Wang et al., 2020; Cai et al., 2021). CIBERSORT, a classic deconvolution algorithm, has been widely used to evaluate TIIC abundance in cancers (Newman et al., 2015). Ge et al. (2019) used CIBERSORT to describe infiltrating features of TIICs and found that M0 & M1 macrophages and CD4 memory activated T cells notably over-infiltrated into colorectal cancer tissues in comparison with normal tissues. In ccRCC, Liu et al. (2020) estimated TIICs using the ESTIMATE algorithm, and found that TIICs have certain effects on genetic mutations and can be used as biomarkers to assess the prognosis of tumor patients. However, for all we know, there was no study on the prognostic value of combined multiple immune cells in ccRCC. As a significant finding in this report, we constructed a novel 4-TIIC signature to predict the prognosis for ccRCC patients based on the levels of Tex, iTreg, Th17, and Tcm. The validation and Cox analysis confirmed the promising prognostic value of this signature in ccRCC.

Tex is a group of T cells with reduced effector functions and continuous expressions of inhibitory receptors such as TIGIT, LAG3, and PDCD1 (Wherry and Kurachi, 2015). Some studies confirmed that the high level of Tex infiltration has an association with deleterious prognosis in various cancers (Kong et al., 2016; Nakano et al., 2018). The infiltration of Tex expressing CD8/PDCD1/TIM3/LAG3 in localized ccRCC can identify the patients with worse prognosis to benefit from adjuvant therapy (Giraldo et al., 2017). iTreg is a highly immunosuppressive, therapy-resistant Treg, which is driven to converse from conventional CD4^+^ T cell, and can suppress the anti-cancer immune reaction, thus enhancing the tumor development (Whiteside et al., 2012). Th17 acts as a pro-inflammatory mediator, which maintains a good balance for regulating suitable adaptive immune reaction through co-operating with the immunosuppressive Treg cells (Marshall et al., 2016). Huang et al. (2019) revealed that long-lived ccRCC patients have high levels of Th17. The last hub TIIC in our signature was Tcm, a subset of memory T cells with key roles in the occurrence and development of cancers (Zhang et al., 2014). The expressional levels of CD4+/CD8+ Tcm were increased in gastric cancer patients after cancer surgery, revealing their tumor-suppressive roles (Zhang et al., 2014). In the current study, we defined the significant prognostic roles of these four TIICs in ccRCC with a novel prognostic signature, namely, Tex and iTreg were risky indicators, while Th17 and Tcm were protective indicators. Moreover, by combining the abundance of these four TIICs, we could exactly predict the prognosis of ccRCC.

Furthermore, given that Tex and iTreg were risky indicators, while Th17 and Tcm were protective indicators, the features of TME in ccRCC patients with high risk should be highly immunosuppressive. The expression levels of PD1, LAG3, and CTLA4 were also upregulated in samples with high risk and could predict poor prognosis in ccRCC. However, PDL1 was not changed in patients with various risks and could not be a prognostic factor in ccRCC. In a previous study, PDL1 expression was observed in 70.4% ccRCC and the corresponding patients had worse prognosis (Kammerer-Jacquet et al., 2017). However, another research revealed that the level of PDL1 expressed in tumor cells was not associated with prognosis in ccRCC (Lee et al., 2020). In conjunction with the conclusion of our study, we hold the opinion that the prognostic value of PDL1 should be further explored.

## Conclusion

To sum up, we analyze the 24 TIIC subgroups in ccRCC patients based on RNA sequencing data using the ImmuCellAI algorithm. We identify that several TIICs are associated with prognosis in ccRCC patients. Thereafter, we identify a 4-TIIC signature, which can accurately predict OS for ccRCC patients. However, it is required to conduct further large-scale clinical investigations to validate our results and explore the potential mechanisms interlinking TIICs in ccRCC progression.

## Data Availability

The original contributions presented in the study are included in the article/Supplementary Material, further inquiries can be directed to the corresponding authors.
