# Supplementary material for: Construction of a novel prognostic signature based on the composition of tumor-infiltrating immune cells in clear cell renal cell carcinoma
Source: Front Genet. 2022 Oct 13;13:1024096. doi: 10.3389/fgene.2022.1024096 (PMC9606472; doi:10.3389/fgene.2022.1024096)
Supplement: Supplementary file 1 [file DataSheet1.DOCX]

**Supplementary Files**


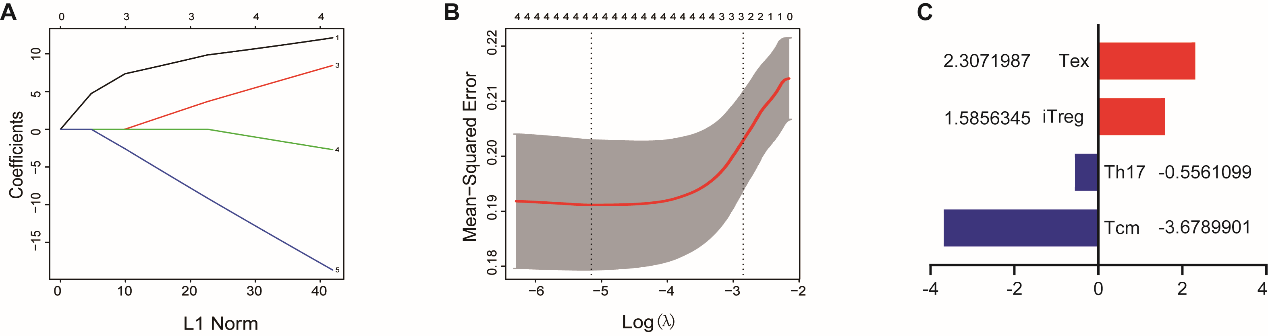


**Figure S1. LASSO Cox regression analysis of TIICs in ccRCC.**

(A) LASSO coefficient profiles of 5 TIICs. (B) 10-fold cross-validations result which identified optimal values of the penalty parameter λ. (C) The distribution of LASSO Cox coefficients in the 4-TIICs signature.

**Table S1. Detailed clinic-pathological information of the HKidE180Su02 cohort.**

| Characteristics | Case |
| --- | --- |
| Gender |  |
| Female | 43 |
| Male | 107 |
| Age |  |
| ≥60 | 63 |
| <60 | 87 |
| Grade |  |
| Well and medium | 103 |
| Poor | 42 |
| Unkonwn | 5 |
| T stage |  |
| T1 | 122 |
| T2 | 17 |
| T3 | 11 |
| N stage |  |
| N0 | 147 |
| N1-2 | 3 |
| Clinical stage | |
| Stage 1 | 122 |
| Stage 2 | 16 |
| Stage 3 | 12 |
| Survival status | |
| Alive | 122 |
| Dead | 28 |

**Table S2. Univariate Cox regression analysis of TIICs infiltration in ccRCC.**

| **TIICs** | **beta** | **HR (95% CI for HR)** | **wald.test** | **P-value** |
| --- | --- | --- | --- | --- |
| CD8.naive | -2 | 0.13 (1.2e-05-1400) | 0.18 | 0.670 |
| Tc | 4.5 | 94 (0.28-32000) | 2.3 | 0.130 |
| Tex | 13 | 350000 (560-2.1e+08) | 15 | **<0.001** |
| Tr1 | 5.7 | 310 (0.26-370000) | 2.5 | 0.110 |
| nTreg | 9.3 | 11000 (13-9900000) | 7.2 | **0.007** |
| iTreg | 7 | 1100 (17-69000) | 11 | **0.001** |
| Th1 | 5.3 | 200 (2.7-15000) | 5.8 | 0.016 |
| Th2 | -3.7 | 0.025 (0.00026-2.4) | 2.5 | 0.110 |
| Th17 | -8.2 | 0.00029 (9.1e-07-0.09) | 7.7 | **0.006** |
| Tfh | 3.8 | 47 (1.2-1800) | 4.3 | 0.039 |
| Tcm | -7.3 | 0.00064 (2.7e-06-0.15) | 6.9 | **0.009** |
| NKT | -0.31 | 0.73 (0.005-110) | 0.01 | 0.900 |
| MAIT | -0.12 | 0.88 (0.00087-890) | 0 | 0.970 |
| DC | -4.6 | 0.01 (8.5e-05-1.3) | 3.4 | 0.064 |
| B.cell | -0.39 | 0.67 (5.8e-06-78000) | 0 | 0.950 |
| Monocyte | 6.5 | 660 (1.3-320000) | 4.2 | 0.040 |
| Macrophage | 2.7 | 15 (0.45-500) | 2.3 | 0.130 |
| NK | 2.2 | 9.1 (0.11-760) | 0.95 | 0.330 |
| Neutrophil | -5.8 | 0.0031 (1.1e-05-0.85) | 4.1 | 0.044 |
| Tgd | -4.6 | 0.0099 (1.5e-05-6.5) | 2 | 0.160 |
| CD4.T | 8.6 | 5400 (5.4-5300000) | 6 | 0.015 |
| CD8.T | 5.4 | 230 (2.5-21000) | 5.6 | 0.018 |
